# Evaluation of Large Language Models in Tailoring Educational Content for Cancer Survivors and Their Caregivers: Quality Analysis

**DOI:** 10.2196/67914

**Published:** 2025-04-07

**Authors:** Darren Liu, Xiao Hu, Canhua Xiao, Jinbing Bai, Zahra A Barandouzi, Stephanie Lee, Caitlin Webster, La-Urshalar Brock, Lindsay Lee, Delgersuren Bold, Yufen Lin

**Affiliations:** 1 Nell Hodgson Woodruff School of Nursing, Emory University, 1520 Clifton Rd NE, Atlanta, GA, 30322, United States, 1 4042514072; 2 Center for Data Science, Emory University, Atlanta, GA, United States; 3 Winship Cancer Institute, Emory University, Atlanta, GA, United States; 4 Department of Medicine, University of Florida, Gainesville, FL, United States

**Keywords:** large language models, GPT-4, cancer survivors, caregivers, education, health equity

## Abstract

**Background:**

Cancer survivors and their caregivers, particularly those from disadvantaged backgrounds with limited health literacy or racial and ethnic minorities facing language barriers, are at a disproportionately higher risk of experiencing symptom burdens from cancer and its treatments. Large language models (LLMs) offer a promising avenue for generating concise, linguistically appropriate, and accessible educational materials tailored to these populations. However, there is limited research evaluating how effectively LLMs perform in creating targeted content for individuals with diverse literacy and language needs.

**Objective:**

This study aimed to evaluate the overall performance of LLMs in generating tailored educational content for cancer survivors and their caregivers with limited health literacy or language barriers, compare the performances of 3 Generative Pretrained Transformer (GPT) models (ie, GPT-3.5 Turbo, GPT-4, and GPT-4 Turbo; OpenAI), and examine how different prompting approaches influence the quality of the generated content.

**Methods:**

We selected 30 topics from national guidelines on cancer care and education. GPT-3.5 Turbo, GPT-4, and GPT-4 Turbo were used to generate tailored content of up to 250 words at a 6th-grade reading level, with translations into Spanish and Chinese for each topic. Two distinct prompting approaches (textual and bulleted) were applied and evaluated. Nine oncology experts evaluated 360 generated responses based on predetermined criteria: word limit, reading level, and quality assessment (ie, clarity, accuracy, relevance, completeness, and comprehensibility). ANOVA (analysis of variance) or chi-square analyses were used to compare differences among the various GPT models and prompts.

**Results:**

Overall, LLMs showed excellent performance in tailoring educational content, with 74.2% (267/360) adhering to the specified word limit and achieving an average quality assessment score of 8.933 out of 10. However, LLMs showed moderate performance in reading level, with 41.1% (148/360) of content failing to meet the sixth-grade reading level. LLMs demonstrated strong translation capabilities, achieving an accuracy of 96.7% (87/90) for Spanish and 81.1% (73/90) for Chinese translations. Common errors included imprecise scopes, inaccuracies in definitions, and content that lacked actionable recommendations. The more advanced GPT-4 family models showed better overall performance compared to GPT-3.5 Turbo. Prompting GPTs to produce bulleted-format content was likely to result in better educational content compared with textual-format content.

**Conclusions:**

All 3 LLMs demonstrated high potential for delivering multilingual, concise, and low health literacy educational content for cancer survivors and caregivers who face limited literacy or language barriers. GPT-4 family models were notably more robust. While further refinement is required to ensure simpler reading levels and fully comprehensive information, these findings highlight LLMs as an emerging tool for bridging gaps in cancer education and advancing health equity. Future research should integrate expert feedback, additional prompt engineering strategies, and specialized training data to optimize content accuracy and accessibility.

## Introduction

More than 18.1 million individuals with a history of cancer were alive in the United States in 2022, and that number is projected to reach 26 million by 2040 [1]. Cancer survivors receive a wide range of treatments, often experiencing severe symptoms or side effects, including fatigue, depression, anxiety, sleep disturbance, pain, cognitive impairment, nausea, vomiting, and neuropathy [2-7]. These symptoms negatively impact survivors’ functional status, quality of life, and overall survival rates [8-11]. Cancer caregivers, typically family members or significant others offering primary emotional and physical support for cancer survivors, experience an array of similar distressing symptoms [12-14]. These symptoms are linked to high caregiving burden, emotional distress, and communication barriers with cancer survivors and providers [15]. In addition, disparities in health care access further exacerbate the challenges faced by cancer survivors and their caregivers, especially those from disadvantaged communities that have limited health literacy or language barriers [16]. Those with limited health literacy and racial and ethnic minorities facing language barriers are at greater risk for poorer access to care [17-19]. Consequently, they tend to experience a heavier symptom burden and poorer health outcomes during and after cancer treatments [20].

With over 3-quarters of the disadvantaged population owning smartphones or computers [21], technology-based intervention programs can bridge the accessibility gap and promote health equity [22,23]. The advent and growth of artificial intelligence have enabled researchers to design tailored and personalized interventions and educational content to meet individual unmet needs [24]. Large language models (LLMs) are advanced artificial intelligence systems that can understand and generate human-like text by training on vast amounts of data [25]. LLMs perform various language tasks, such as answering questions and translating languages. How questions are asked can significantly affect the performance of LLMs. This process, known as prompt engineering, is crucial for obtaining accurate and relevant responses from LLMs [26,27]. While LLMs have demonstrated remarkable potential in cancer research [28-31], their efficacy in real-world scenarios, such as cancer care and education, which often require advanced levels of comprehension, have yet to be thoroughly assessed.

Recent advancements in LLMs, such as GPT-4 and GPT-4 Turbo (OpenAI) [32,33], have demonstrated their exceptional proficiency in completing various tasks, including coding, design, and content summarization. Previous research [34,35] indicates that LLMs can capture large volumes of text effectively, even without specialized domain knowledge. This ability highlights its sophistication in processing and understanding information across a broad spectrum of topics, and its potential to significantly aid in analyzing unstructured data in clinical environments (eg, clinical notes) [34,35]. However, there are several notable gaps in the current knowledge. First, while LLMs have demonstrated high levels of accuracy in understanding extensive texts [34,36,37], even minor inaccuracies can have detrimental effects on patient outcomes [38], particularly regarding actionable advice. Therefore, the content they generate still necessitates additional expert verification to ensure it is error-free and ready to be presented to patients and their caregivers. Second, although previous research [36,37] has demonstrated promising results in content summarization, these LLMs are often not applied in clinical environments, or they specifically address cancer care and education among disadvantaged groups that has limited health literacy or language barriers [39]. Finally, most educational resources for cancer care are available exclusively in English, which can create comprehension challenges for non-English speakers (eg, Hispanic individuals and immigrants). Also, cancer survivors and their caregivers, already overwhelmed by treatment, often lack the time to read lengthy content. Therefore, it is essential to provide educational content in multiple languages and in concise content to ensure effective communication and education [40].

To address these gaps, our team aimed to evaluate how LLMs perform in tailoring educational content to enhance accessibility and comprehension for cancer survivors and their caregivers. In this study, our primary task was to evaluate and compare the capabilities of multiple GPT-based LLMs in generating concise, low-literacy-level, and multilingual educational content tailored for cancer survivors and their caregivers with limited health literacy or language barriers. Specifically, we aimed to evaluate the overall performance of LLMs in generating tailored educational content that adheres to a strict word limit, a sixth-grade reading level, and high-quality criteria (clarity, accuracy, relevance, completeness, and comprehensibility), compare the performances of 3 GPT models (GPT-3.5 Turbo, GPT-4, and GPT-4 Turbo), and explore how different prompt structures (textual vs bulleted format) influence the quality of the generated content. This approach helps them manage their symptoms more effectively, thereby reducing health disparities and promoting health equity.

## Methods

### Design

This study involved a multistep methodology that included: (1) specifying the exact task requirements for the LLMs, to produce educational content on 30 selected cancer care topics written at a sixth-grade reading level, limited to 250 words, and translated into Spanish and Chinese; (2) generating tailored educational content using 3 GPT models (GPT-3.5 Turbo, GPT-4, and GPT-4 Turbo) with 2 prompt styles (textual and bulleted); (3) expert evaluation of the generated content’s adherence to word count, reading level, and 5 quality criteria; and (4) statistical analyses (ANOVA [analysis of variance] and chi-square test) to compare performance across models and prompt formats.

### Prompt Engineering

To promote the accessibility and comprehension of educational content for cancer survivors and their caregivers with limited health literacy and language barriers, we structured prompts to have LLMs produce content at a low reading level, maintain a word limit of 250, and provide Spanish and Chinese translations for each topic, as described below [41].

The Flesch-Kincaid Grade Level (FKG) system [42] was used to assess the readability of content produced by the LLMs. The FKG level is a readability test designed to indicate how difficult a text is to understand. It calculates the grade level required for someone to comprehend the text. The FKG is based on word length and sentence length, providing a numerical score that corresponds to US grade levels [42]. The National Institutes of Health (NIH) and the American Medical Association (AMA) suggest that patient education materials should be written at a reading level no higher than the sixth grade [43]. This recommendation is in place to guarantee that the information is reachable by a broad spectrum of individuals, encompassing those with limited health literacy. Therefore, our research targets an FKG level of 6 to align with this guidance.

We set a 250-word limit for our educational content, recognizing that cancer survivors and their caregivers are frequently preoccupied with treatment schedules and daily responsibilities, leaving them with limited time for reading [44]. This word limit is designed to ensure that participants can complete the reading within 5 minutes, making the task both manageable and feasible within their schedules.

Furthermore, it is shown that prompts exert a considerable impact on the responses generated by LLMs [45]. Therefore, we compared different prompts, including both textual and bulleted formats, to determine which approach yields better results.

The prompts we used are as follows:

1. Textual format: “Please summarize the following content in Flesch-Kincaid Grade level of 6 and under 250 words: [original text]”
2. Bulleted format: “Please summarize the following content into bullet points in Flesch-Kincaid Grade level of 6 and under 250 words: [original text]”
3. Spanish translation: “Please translate the following content into Spanish: [tailored text]”
4. Chinese translation: “Please translate the following content into Chinese: [tailored text]”

### Expert Evaluation

**Figure 1.**
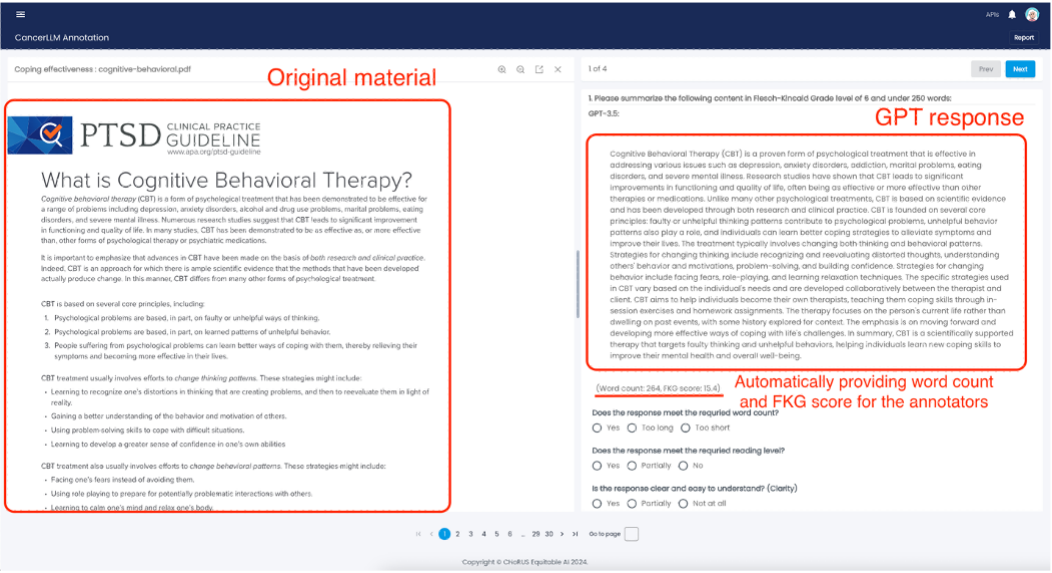
A screenshot of Cohort Adjudication and Data Annotation application.

We assembled a panel of 9 oncology experts, comprising 4 oncology professors, 4 doctoral students, and 1 medical resident. Among them, all are fluent in English, with 4 experts proficient in Chinese and 1 proficient in Spanish. Each response generated by the LLMs was evaluated by at least 2 experts to ensure a comprehensive assessment, except for the Spanish translation task, which was evaluated by a single expert. The panel conducted several Zoom meetings: the initial meeting provided training on content evaluation, and 3 additional meetings were held to discuss the results and feedback. Each expert was assigned 10 topics to evaluate and was required to provide feedback on the errors committed by the LLMs. These experts critically reviewed and annotated the LLM-generated content using a web-based Cohort Adjudication and Data Annotation (CADA) application [34] (Figure 1) developed by our team.

### Data Sources

Our primary sources for content generation were cancer survivors and caregiver education materials from the National Cancer Institute and the National Comprehensive Cancer Network guidelines [46,47]. We selected 30 distinct topics covering a range of content such as fatigue, depression, anxiety, pain, cognitive impairment, nutrition, physical activity, healthy lifestyle, family communication, coping skills, and more. The selection of topics was informed by insights from our previous qualitative interviews with cancer survivors and their caregivers [48] and an extensive review of the literature [49-51]. We identified the key areas of interest and specific needs of cancer survivors and their caregivers with limited health literacy or language barriers, resulting in these 30 topics.

### Appraisal Criteria

Based on a previous study of evaluating responses from LLMs [34], we formulated a set of multidimensional criteria to thoroughly assess the performance of LLMs, which include adherence to a word limit of 250 words, achieving a reading level as per the FKG of below 6, and quality assessment: (1) clarity (ie, ease of understanding in the response); (2) accuracy (ie, the response does not contain errors, like medical or language errors, that could negatively impact patients and their caregivers); (3) relevance (ie, the response is fully grounded in the materials we provided); (4) completeness (ie, the response encompasses all critical points from the materials); (5) comprehensibility (ie, the response is understandable that readers can apply it to their daily routine).

In terms of word limit, “yes” refers to a word limit within 250 words, and “no” refers to a word limit of more than 250 words. The reading level was evaluated using “yes” for an FKG level ≤6; “partial” for an FKG level of 6 to ≤8; and “no” for an FKG level >8). The FKG level was calculated by the Python package Textstat (version 0.7.3, Azu). For the quality assessment criteria, we implemented a scoring system in which evaluations were quantified based on their alignment with the expected outcomes. A score of 2 was assigned for “yes” evaluations, indicating full compliance; a score of 1 was given for “partial” evaluations, reflecting partial compliance; and a score of 0 was allocated for “no” evaluations, indicating noncompliance. The quality assessment included 5 criteria (1-5), each contributing a maximum of 2 points, for a total possible score of 10. The overall quality assessment ranged from 0 to 10, with 0 representing the absence or lowest quality and 10 indicating the highest quality. For translation tasks, “yes” indicates a completely accurate translation, “partially” refers to a generally correct and understandable translation with minor errors, and “no” refers to a completely inaccurate translation containing incorrect or misleading information. Accuracy scores are calculated as the proportion of evaluations labeled as “yes.”

### Data Analysis

Descriptive analyses were conducted to determine the frequencies, percentages (for word limit, reading levels, and translations), mean and SDs (for quality scores) of major variables. Quality scores were determined by calculating the mean scores for each criterion and then obtaining the overall scores through their summation. To compare the differences in each model or prompt, we used ANOVA or chi-square tests, as applicable. Values of *P*<.05 were considered to indicate a significant level. All analyses were conducted using Python statistical packages.

### Ethical Considerations

The study protocol (STUDY00004750) was approved with exemptions by the institutional review board at Emory University. Oral consent was obtained from 9 oncology experts, as no protected health information was collected. All participants were informed of the voluntary nature of their participation and their right to withdraw at any time without consequence. No protected health information or personally identifiable information was collected, and all research data were anonymized to maintain confidentiality. Study materials were securely stored and accessible only to authorized research team members. Participants did not receive any monetary or nonmonetary compensation for their involvement. The study was conducted in accordance with the US Common Rule (45 CFR 46) [52].

## Results

### Overall Performance of Large Language Models

In this study, 360 annotation values were collected from 9 experts. Overall, LLMs have shown excellent performance in tailoring content based on our criteria. For word limit, 267/360 responses (74.2%) were within the word limit (less than 250 words) set for the task. The result indicates the excellent ability of LLMs to produce responses that adhere to specified word limit requirements. Regarding reading levels, LLMs demonstrated moderate performance, with 105/360 responses (29.2%) fully meeting the specified FKG level (FKG level ≤6), 107/360 (29.7%) being partially satisfactory (FKG level of 6‐8), and 148/360 (41.1%) not aligning with the provided FKG level (FKG level >8).

LLMs demonstrated consistently high average scores across all quality criteria (total score: 8.933 out of 10). The highest average score achieved was 1.91 on relevance, highlighting the LLMs’ ability to generate content that was highly pertinent to the given prompts. The lowest average score observed was 1.58 out of 2 in the category of completeness, indicating a moderate adherence to providing responses that capture all key points. In the translation tasks, the LLMs demonstrated high performance, with 87/90 accuracy translations (88%) for Spanish and 73/90 (81%) for Chinese translation.

### Three Generative Pretrained Transformer Models Comparisons: GPT-3.5 Turbo, GPT-4, and GPT-4 Turbo

GPT-4 demonstrated a superior capability in adhering to the specified word limit, with 101/120 responses (84.2%) falling within 250 words (Table 1). In contrast, GPT-3.5 Turbo and GPT-4 Turbo exhibited a relatively lower proficiency, with 86/120 (71.7%) and 80/120 (66.7%) responses meeting the word limit, respectively. As shown in Table 2, when comparing the models based on word limit, the chi-square test demonstrated a significant difference among the three models (*P*=.006).

**Table 1.** Performance of all models and prompts on the summarization task.

	GPT^a^-3.5 Turbo^b^ ^c^			GPT-4^b^ ^c^			GPT-4 turbo^b^ ^c^		
	Total	Textual format	Bullet points	Total	Textual format	Bullet points	Total	Textual format	Bullet points
Word limit, %	71.7 (86/120)	46.7 (28/60)	96.7 (58/60)	84.2 (101/120)	91.7 (55/60)	76.7 (46/60)	66.7 (80/120)	51.7 (31/60)	81.7 (49/60)
Reading level, %	23.3 (28/120)	18.3 (11/60)	28.3 (17/60)	21.7 (26/120)	21.7 (13/60)	21.7 (13/60)	42.5 (51/120)	53.3 (32/60)	31.7 (19/60)
Accuracy, mean (SD)	1.775 (0.493)	1.767 (0.5)	1.783 (0.49)	1.767 (0.561)	1.8 (0.48)	1.733 (0.634)	1.783 (0.522)	1.8 (0.48)	1.767 (0.563)
Clarity, mean (SD)	1.792 (0.447)	1.833 (0.418)	1.75 (0.474)	1.833 (0.396)	1.867 (0.389)	1.8 (0.403)	1.8 (0.422)	1.883 (0.324)	1.717 (0.49)
Relevance, mean (SD)	1.892 (0.362)	1.883 (0.415)	1.9 (0.303)	1.925 (0.295)	1.883 (0.372)	1.967 (0.181)	1.925 (0.264)	1.9 (0.303)	1.95 (0.22)
Completeness, mean (SD)	1.558 (0.632)	1.533 (0.623)	1.583 (0.645)	1.575 (0.617)	1.483 (0.624)	1.667 (0.601)	1.617 (0.582)	1.583 (0.619)	1.65 (0.547)
Comprehensibility, mean (SD)	1.808 (0.436)	1.817 (0.469)	1.8 (0.403)	1.892 (0.312)	1.883 (0.324)	1.9 ( 0.303)	1.858 (0.35)	1.9 (0.303)	1.817 (0.39)
Total score, mean (SD)	8.825 (1.643)	8.833 (1.748)	8.817 (1.546)	8.992 (1.247)	8.917 (1.239)	9.067 (1.26)	8.983 (1.195)	9.067 (1.087)	8.9 (1.298)

a GPT: Generative Pretrained Transformer.

b The performance (%) of GPT-3.5 Turbo was 93.3% (28/30), GPT-4 was 96.7% (29/30), and GPT-4 Turbo was 100% (30/30) for the Spanish translation. The overall performance (%) of the three GPT models in Spanish translation was 96.7% (87/90).

c The performance (%) of GPT-3.5 Turbo was 76.7% (23/30), GPT-4 was 86.7% (26/30), and GPT-4 Turbo was 80% (24/30) for the Chinese translation. The overall performance (%) of the three GPT models in Chinese translation was 81.1% (73/90).

**Table 2.** Statistical analysis results from analysis of variance and chi-square tests.

Group and criterion	PR(>F)^a^	Chi-square (*df*)
**Models**		
Accuracy	0.97	—^b^ (2)
Clarity	0.721	— (2)
Relevance	0.63	— (2)
Completeness	0.748	— (2)
Comprehensibility	0.215	— (2)
Total score	0.572	— (16)
Word limit	0.006	10.178 (2)
Reading level	<0.001	35.468 (4)
Spanish translation	0.355	2.069 (2)
Chinese translation	0.602	1.015 (2)
Translation	0.481	1.463 (2)
**Prompts**		
Accuracy	0.213	— (2)
Clarity	0.028	— (2)
Relevance	0.177	— (2)
Completeness	0.154	— (2)
Comprehensibility	0.149	— (2)
Total score	0.939	— (8)

a PR(>F): probability that the F-statistic is greater than the observed value under the null hypothesis.

b —: not applicable.

Regarding the assessment of reading level, GPT-4 Turbo met the required FKG level of 6 in 51/120 (42.5%) cases, nearly doubling the performance of the other 2 models: 26/120 (21.7%) for GPT-4 and 28/120 (23.3%) for GPT-3.5 Turbo. The result indicated significant discrepancies among the models in adherence to the specified reading level (*P*<.001), with GPT-4 Turbo performing better compared with the other 2 models.

**Figure 2.**
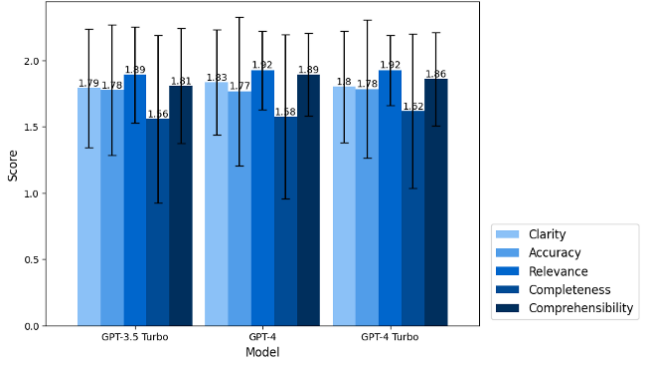
Assessment scores on each criterion between different models. GPT: Generative Pretrained Transformer.

In terms of quality assessment, each of the LLMs attained a high score exceeding 8.8 out of 10, with GPT-4 and GPT-4 Turbo achieving 8.992 and 8.983, respectively, and GPT-3.5 Turbo trailing slightly at 8.825. Upon evaluation of each criterion, the performance of all models was found to be similar (Figure 2). The application of ANOVA tests to each criterion revealed no significant differences among the 3 models (*P*=.57).

In the translation tasks, GPT-4 Turbo exhibited perfect accuracy with a 30/30 (100%) success cases in Spanish translation, whereas GPT-4 and GPT-3.5 Turbo exhibited slightly lower, yet commendable success rates of 29/30 (97%) and 28/30 (93%), respectively. For the Chinese translation task, GPT-4 outperformed the other models with an accuracy of 26/30 (87%). In contrast, GPT-3.5 Turbo and GPT-4 Turbo achieved 23/30 (77%) and 24/30 (80%), respectively. The 3 models did not show a significant difference in the translation task (*P*=.48).

### Two Different Prompt Comparisons: Textual and Bulleted Formats

**Figure 3.**
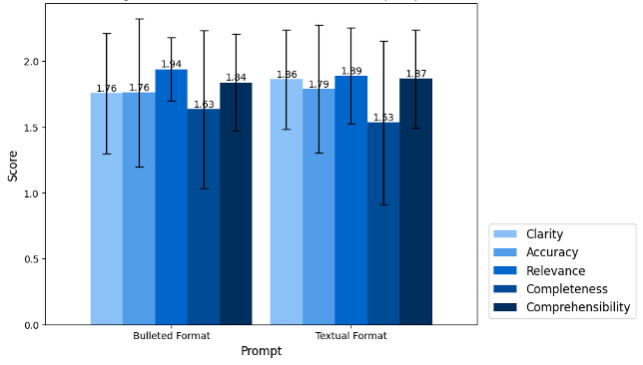
Assessment scores on each criterion between different prompts.

We compared 2 prompting methods in terms of word limits, reading level, and quality assessment. The major difference noted in the comparison of the 2 prompts was that responses generated from prompt 2 (bulleted format) were superior in adhering to the target word limit. Specifically, 153/180 responses (85%) from prompt 2 successfully achieved the word limit, in contrast to 114/180 responses (63.3%) from prompt 1 (textual format) that fully satisfied the word limit. Using prompt 1 resulted in only 56/180 responses (31.1%) meeting our desired reading level, with a slight decrease to 49/180 (27.2%) for prompt 2. For the 5 quality criteria, both prompts achieved high scores (Figure 3). Upon performing an ANOVA test to assess the differences in performance between the 2 prompts (Table 2), it was found that the variations between them were not significant (*P*=.939). However, the 2 prompt formats demonstrated a significant difference in the clarity criterion (*P*=.03).

### Error Analysis

The errors that LLMs committed were categorized into inaccurate scope, inaccurate definition, inaccurate expression, meaningless points, and inaccurate word. Some examples are shown in Table 3.

**Table 3.** Error cases and analysis.

Model	Topic	Output	Error type	Reason
GPT-3.5 Turbo	Nutrition	“It advises limiting animal-based food, processed food, and alcohol consumption.”	Inaccurate scope	The chapter only mentions to limit red meat, not all animal-based foods (says it can make up half or less of diet).
GPT-3.5 Turbo	Sexual Health Issues in Men with Cancer	“It is still important to maintain intimacy with a partner.”	Inaccurate expression	The tailored content sounds a little judgmental whereas the original document says, “probably still important” and is less assuming.
GPT-3.5 Turbo	Relaxation	“多喝液体”	Inaccurate word	Based on the English sentence: " Drinking plenty of liquids”, “liquids” can be better translated into “水.”
GPT-4	Mindfulness	“These practices involve focusing the mind on present sensations, such as breathing, a sound, or an image.”	Inaccurate definition	It seems to define meditation and mindfulness in one overarching definition, which only defines meditation. The model merged definitions of MBSR^a^ and MBCT^b^ together and did not include difference between types.
GPT-4	Family Communication	“El apoyo de la comunidad podréda ser beneficioso durante este difícil período.”	Inaccurate word	Based on the English sentence: “Support from the community might be beneficial during this difficult period. “, “difícil período” should be “período difícil”
GPT-4 Turbo	Making a Difference	“Learning: Educating yourself about cancer can empower you to assist others. Resources are available online, by phone, and in print.”	Meaningless point	The customized content falls short in terms of actionability. The purpose of tailoring content is to educate patients and caregivers, rather than expecting them to educate themselves.

a MBSR: Mindfulness-based stress reduction.

b MBCT: Mindfulness-based cognitive therapy.

A common error observed with LLMs is their tendency to integrate their own knowledge and interpretation rather than adhering strictly to the provided materials, such as an inaccurate scope. For instance, when the text specified “to limit red meat.*”* in the Nutrition topic, GPT-3.5 Turbo inaccurately generalized this advice to “limiting animal-based food.” This interpretation is not entirely correct, as animal-based food encompasses more than just red meat, including white meat such as chicken, which the original material did not intend to restrict.

Other observed errors involve inaccurate expressions. For instance, in the Sexual health issues in men with cancer topic, the original content suggested, *“*It is probably still important to maintain intimacy with a partner.*”* However, GPT-3.5 Turbo revised this to “it is still important to maintain intimacy with a partner.” This alteration results in a tone that may seem judgmental, deviating from the original’s more tentative stance.

An example of inaccurate definition was identified within the Mindfulness topic, where GPT-4 defined meditation and mindfulness in one overarching definition for meditation. It also merged definitions of mindfulness-based stress reduction and mindfulness-based cognitive therapy without highlighting differences between the mindfulness interventions.

LLMs may also include information that, while accurate, might not be actionable for patients. For instance, in the Making a difference topic, GPT-4 Turbo correctly sourced from the material that “Learning: Educating yourself about cancer can empower you to assist others. Resources are available online, by phone, and in print.” However, this information becomes less useful in the absence of specific links or directions that could guide patients on where to start their education.

Finally, with respect to translation quality, the primary error observed related to inaccurate word choice. In particular, when an English term offers multiple potential translations, LLMs often encounter difficulty in selecting the most contextually appropriate option. For example, in the Relaxation topic, GPT-3.5 Turbo translated “多喝液体" as “drink more liquids.” Although “液体" does literally translate to “liquids,” the more natural and contextually appropriate term would be “水.”

## Discussion

### Principal Findings

To our knowledge, this is the first study to evaluate the capability of LLMs in tailoring educational content for cancer survivors and their caregivers with limited health literacy or language barriers. In our study, all 3 LLMs have demonstrated overall excellent performance in most criteria. The more advanced GPT-4 family models showed better overall performance compared with GPT-3.5 Turbo. GPT-4’s high adherence to word limits and GPT-4 Turbo’s better compliance to reading level compliance proved their ability to meet our requirements when tailoring content. Prompting GPTs to produce bulleted-format content is likely to result in better educational content compared with textual-format content. All models exhibit strong capability in generating highly relevant content. However, they fall short in terms of completeness. Overall, it is proven that LLMs are highly effective in tailoring, condensing, and translating educational content for cancer survivors and their caregivers with limited health literacy or language barriers. These findings inform future versions of LLMs to focus more on the reading level and completeness of their output and the development of tailored intervention materials for cancer survivors and their caregivers. These promising results also indicate that LLMs can be a valuable tool in making educational content more accessible and comprehensible to diverse patient populations.

The capabilities of LLMs in text analysis have been well studied. For example, our previous study [34] examined the potential of LLMs to categorize clinical concepts from patient notes. Yet, this study focused solely on the LLMs’ comprehension of patients’ conditions from clinical notes rather than educational content. Study by Veen et al [53] assessed approaches for LLMs to summarize clinical texts. Although it demonstrated overall preferred performance, especially GPT-4, over human experts, the study was limited to the summarization of radiology report findings and confined to 3 attributes: completeness, correctness, and conciseness, whereas our study expanded on this topic by evaluating LLMs against 7 distinct criteria. Furthermore, none of the existing studies focus on education regarding supportive care in cancer, whereas our innovative findings make a significant contribution to the literature in this field.

Despite the excellence of LLMs in adhering to specified word limits and generating high-quality content, several challenges remain. One notable area where LLMs struggle is in adjusting the reading level of the content to accommodate patients from various educational levels. The content tailored by LLMs often does not meet the intended FKG level. This oversight implies that some individuals might find the content overly complex, potentially hindering their understanding of health information and educational content [54,55]. Addressing this challenge is essential for maximizing the applicability of LLMs and ensuring that all cancer survivors receive the support they need to manage their cancer effectively. In future work, in-context learning could be used to offer more detailed guidance to LLMs, focusing on the potential vocabularies frequently appeared in content exceeding the specified FKG level of 6. In addition, retrieval-augmented generation could be implemented to embed vocabularies aligned with an FKG level of 6, thereby enhancing the model’s performance.

It is also observed that the accuracy of Spanish translations is significantly higher than that of Chinese translations. This finding is expected, given the abundance of Spanish content available on the internet compared with Chinese content that can serve as training materials. Previous studies [56,57] have shown that LLMs’ performance in different languages has a clear correlation with the proportion of each language in the pretraining corpus. Without fine-tuning, LLMs have a much higher performance in high-resource languages like German, French, and Spanish, and a significantly lower performance in low-resource languages like Kannada, Occitan, and Western Frisian [56,57]. In future work, integrating high-quality bilingual medical corpora that includes parallel texts of patient education materials, clinical guidelines, and culturally tailored health information could be a promising approach. Fine-tuning LLMs on such specialized corpora may provide them with domain-specific vocabulary and context, thereby increasing their ability to produce accurate, culturally sensitive translations.

The educational content errors could be detrimental to cancer survivors and their caregivers by providing false physical activity, diet, or medication suggestions. Therefore, content produced by LLMs should undergo thorough evaluation and validation before the content is used in a clinical setting [38,58,59]. Our analysis has identified multiple errors in the outputs from LLMs, including inaccuracies in scope, expression, and definition. These types of errors can lead to the dissemination of misinformation, potentially causing harm to patients [60]. Therefore, such inaccuracies must be identified, analyzed, and rectified to prevent any negative impacts on patient care. Our study also detected some meaningless points that were not actionable in LLMs’ outputs, which could increase the reading burden on patients and their caregivers. Recommendations should highlight actionable information for cancer survivors and their caregivers to reduce the burden of reading educational content, emphasizing the need for LLMs to prioritize the use and applicability of the information they present. In addition, education content should be evaluated and validated by content experts before the it is available to cancer survivors and their caregivers.

In addition, both Xiao et al’s and Asthana et al’s studies [36,37] evaluated the performance of fine-tuned LLMs in nonclinical environments. Their results highlighted the significant potential of LLMs in summarizing general text through the adoption of advanced fine-tuning techniques. It is possible that fine-tuning could further improve LLMs’ capacity to analyze educational content specifically tailored for groups such as cancer survivors and their caregivers with limited health literacy or language barriers. With this additional data, more advanced fine-tuning techniques such as instruction tuning [57,61,62] and parameter-efficient fine-tuning [63] can be implemented, and are likely to further enhance the performance.

### Limitations

While the study has shown promising results, it has several limitations. First, the dataset size remains relatively small, which could restrict the generalizability of the findings to broader topics. Second, we lacked participant assessment. Relying solely on oncology experts to evaluate the outputs from LLMs might create obstacles when applying these findings to actual cancer patients and their caregivers. While our oncology experts deeply value caring for disadvantaged populations with limited health literacy or language barriers, it’s important to note that they are highly educated and might have unintentional biases. This could make it challenging for them to view educational content from the perspective of individuals with low health education and literacy. Therefore, future studies can be broadened to include a wider range of educational topics and additional annotations from cancer patients and their caregivers. Third, this study was limited to zero-shot learning because of the lack of training data. It could be expanded by collecting tailored content from human experts to serve as training data to incorporate few-shot learning and fine-tuning techniques. In addition, chain-of-thought reasoning and in-context learning also present promising avenues for future exploration, particularly because they do not rely on additional training data. Finally, due to a limited number of annotators from diverse backgrounds, our study was only able to evaluate translations in 2 languages. Our analysis suggests that translation performance can vary between languages, influenced by the availability of content in each language. It is important to note that these findings may not be generalizable to languages spoken by smaller populations, where content availability and linguistic nuances could further affect translation accuracy. In future research, more extensive evaluations of translation tasks involving other languages, especially low-resource languages, should be conducted to expand the applicability.

### Conclusions

The study highlights the application of LLMs in cancer care while being cognizant of their potential limitations. All 3 LLMs have demonstrated overall high capability in tailoring educational content for cancer survivors and their caregivers with limited health literacy or language barriers. GPT-4 family models showed better overall performance compared with GPT-3.5 Turbo. Prompting GPTs to produce bulleted-format content can generate better educational content. The findings from this study inform the intervention development and implementation in cancer symptom management and health equity. Additional studies are warranted to expedite the integration of AI-driven solutions into clinical settings.

## Abbreviations

AMA: American Medical Association
ANOVA: analysis of variance
CADA: Cohort Adjudication and Data Annotation
FKG: Flesch-Kincaid Grade Level
GPT: Generative Pretrained Transformer
LLM: large language model
NIH: National Institute of Health
